# The hydrolysis of geminal ethers: a kinetic appraisal of orthoesters and ketals

**DOI:** 10.3762/bjoc.12.143

**Published:** 2016-07-15

**Authors:** Sonia L Repetto, James F Costello, Craig P Butts, Joseph K W Lam, Norman M Ratcliffe

**Affiliations:** 1 Faculty of Applied Sciences, University of the West of England, Bristol, BS16 1QY, UK; 2 School of Chemistry, University of Bristol, Bristol, BS8 1TS, UK; 3 Airbus Operations Ltd., Filton, Bristol, BS34 7PA, UK

**Keywords:** FDII, geminal ethers, kinetics

## Abstract

A novel approach to protecting jet fuel against the effects of water contamination is predicated upon the coupling of the rapid hydrolysis reactions of lipophilic cyclic geminal ethers, with the concomitant production of a hydrophilic acyclic hydroxyester with de-icing properties (Fuel Dehydrating Icing Inhibitors - FDII). To this end, a kinetic appraisal of the hydrolysis reactions of representative geminal ethers was undertaken using a convenient surrogate for the fuel–water interface (D_2_O/CD_3_CN 1:4). We present here a library of acyclic and five/six-membered cyclic geminal ethers arranged according to their hydroxonium catalytic coefficients for hydrolysis, providing for the first time a framework for the development of FDII. A combination of ^1^H NMR, labelling and computational studies was used to assess the effects that may govern the observed relative rates of hydrolyses.

## Introduction

**Scheme 1:**
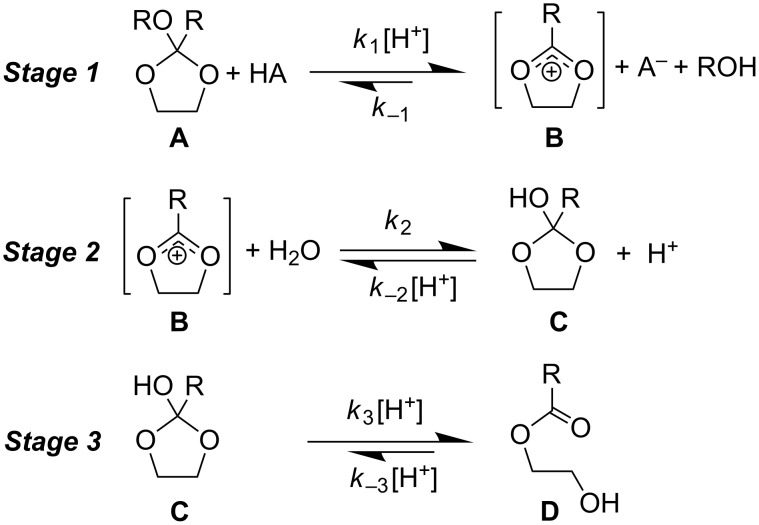
The three-stage mechanism for the specific acid-catalysed hydrolysis of cyclic orthoester **A**.

Our interest in organic dehydrating agents for applications in the aeronautical industry is stimulated by the commercial case for developing a new approach to managing water contamination in jet fuel. The acid-catalysed hydrolysis reactions of cyclic orthoesters present an attractive platform from which to develop Fuel Dehydrating Ice Inhibitors (FDII) [1], since jet fuel is itself mildly acidic [2] and the products of hydrolysis can in principle afford protection against ice formation by residual water [3]. From Brønsted’s ground-breaking work on acid catalysis [4], to more recent investigations as models for glycosidic bond cleavage [5], orthoesters have been examined using a range of methods, solvents (i.e., water, methanol, dioxane, and mixtures thereof), and conditions [6–7]. It has been difficult to draw upon this disparate body of data for our purposes, as potential FDII will operate under non-buffered, relatively apolar conditions. Though easily stated, the mechanism for the formation of a charged intermediate [5,7–11] followed by attack of water, cleavage of RO–C bonds, and several proton-transfer reactions is non-trivial [6,8,10,12–13]; and indeed the debate about the factors influencing the overall rate of reaction and the synchronicity of steps has yet to achieve consensus [11,14–15]. Factors such as solvent [13], catalyst p*K*_a_ [9], –OR basicity [6,16–17], the kinetic anomeric effect [18], the impact of substituents upon the formation [10–11,19–22] and reactivity of charged intermediates [14] have all attracted scrutiny. A generic 2-alkoxy-2-alkyl-1,3-dioxolane **A** is used to illustrate the generally accepted specific acid-catalysed three-stage hydrolysis mechanism of orthoesters (Scheme 1) [23].

The first stage sees the generation of 1,3-dioxolan-2-ylium cation **B** along with alcohol; as water is usually in excess the reverse reaction with ROH (i.e., *k*_−1_) is considered negligible. It has been established that cyclic orthoesters, and in particular those derived from 1,3-dioxolanes, initially undergo exclusive cleavage of the exocyclic alkoxy group [19]. In reviewing the case of orthoesters, Kresge et al. suggests two mechanistic extremes [19,24]. A concerted catalysed process, wherein the –OR group undergoes protonation as the C···O(H^+^)R bond begins to undergo cleavage. The alternative stepwise process involves the C–OR bond spontaneously cleaving at a rate similar to that of RO^−^ protonation by H_3_O^+^. The latter describes a spontaneous uncatalysed hydrolysis. In the case of 1,3-dioxolanes, the concerted mechanism is believed to dominate [25]; however Guthrie [9] asserts that orthoesters are in fact delicately poised between stepwise and concerted processes; depending upon the substituent, both mechanisms are operational for aryl dimethyl orthoformates [21]. Stage 2 sees reaction of **B** with H_2_O to afford 2-hydroxy-1,3-dioxolane **C** (i.e., *k*_2_) with the overall equilibrium constant *K*_2_ = *k*_2_/*k*_−2_[H^+^]. Finally, stage 3 sees the acid-catalysed (i.e., *k*_3_[H^+^]) cleavage of **C** to afford hydroxy ester **D**.

**Figure 1:**
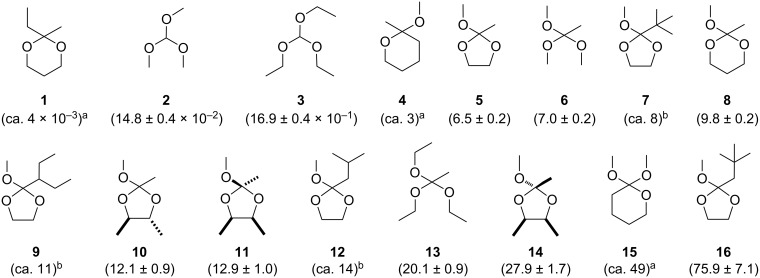
Hydroxonium catalytic coefficients (*k*_H+_ M^−1^ s^−1^ including standard errors where appropriate) for **1–16** as determined in this study. ^a,b^Values of *k*_H+_ determined via calibration with respect to **8** [18] and **16** [23], respectively (see Experimental section).

For acyclic geminal ethers stage 1 is invariably rate limiting, i.e., *k*_3_ > *k*_1_. For cyclic systems *k*_−3_ becomes more dominant in the pH range of about 4–6 [23], however stage 1 remains rate limiting [26]. The overall rate of reaction can therefore be established by measuring the consumption of the geminal ether [27]. We present here kinetic data measured for a range of acyclic orthoformates, orthoacetates, 1,3-dioxolane orthoesters, oxanes, and 1,3-dioxanes (Figure 1), and consider the factors which may modulate the rates of hydrolyses.

## Results and Discussion

The ratio of rates corresponding to the hydrolysis of some six-membered ketals and orthoesters were reported previously [18]. Employing a similar approach using the same solvent system (i.e., D_2_O/CD_3_CN 1:4 v/v), quantitative rate data for the hydrolysis reactions of acyclic and cyclic derivatives **1–16** were determined, affording a library of potential FDII, organised unambiguously by hydroxonium catalytic coefficient *k*_H+_ (Figure 1, *k*_H+_ M^−1^ s^−1^ in parentheses; see Supporting Information File 1 for further details). The observed rate constants (*k*_obs_) were determined using the rate equation integrated over time, using ^1^H NMR spectroscopy to evaluate relative concentrations at 25 ± 0.5 °C. Having already established [1] that specific acid catalysis is more likely to be the dominant mechanism operating in jet fuel/water mixtures, the complications attending the use of buffered systems (and potential *k*_3_ catalysis by A^−^) are circumvented by using mineral acid (i.e., HCl, where [H^+^] was confirmed via the measurement of [Cl^−^] using ion chromatography). The observed rate constant for hydrolysis increases linearly with increasing catalyst concentration, i.e., *k*_obs_ = *k*_H+_ [H^+^]. Nevertheless, we have used the same acid catalyst concentration ([H^+^] = 2 × 10^−4^ M) as previous workers [18] except in the case of particularly slow reactions where [H^+^] was increased to 5 × 10^−4^ M to ensure completion within a reasonable time.

### Acyclic orthoesters

Previous workers compared the rates of hydrolysis for cyclic **1**, **4**, **8** and **15** as determined in D_2_O/CD_3_CN (1:4 v/v) with acyclic derivatives whose rates were established in water [18]. Further, the literature suggests that acyclic **3** and **6** hydrolyse at similar rates (i.e., *k*_H+_ = 1.4 and 1.2 × 10^4^ M^−1^ s^−1^, respectively) [4,7] which is surprising given the charge stabilisation expected to accompany the replacement of an H atom with a methyl group. A comparison of the relative rates of pairs of acyclic orthoesters (i.e*.,* **2** + **3**, **2** + **6**, and **3** + **13**) was performed to address such inconsistencies. An eleven-fold increase in the hydroxonium catalytic coefficient accompanies the replacement of the MeO- with EtO- moieties in orthoformates **2** → **3** (Figure 1). A three-fold increase in *k*_H+_ accompanies MeO → EtO for orthoacetates **6** → **13**. As expected, alkyl substituents on the carbonyl carbon atom have a greater accelerating influence on the overall rate of hydrolysis (H → Me ca. 50-fold) compared to the ethereal substituent (Me → Et 3–11-fold), with the fastest rate of hydrolysis for acyclic systems considered here being achieved by **13** (*k*_H+_ = 20.1 ± 0.9 M^−1^ s^−1^).

### The relative reactivity of six-membered cyclic ketals and orthoesters

The ratio of rates for hydrolysis (as opposed to *k*_H+_) for **1**, **4**, **8** and **15** have been reported previously [18]. We prepared **8** and evaluated *k*_H+_ using the same conditions employed by these workers to calibrate values of *k*_H+_ for **1**, **4**, and **15** (Figure 1). Acyclic **6** was also examined alongside **8** to correlate the hydroxonium catalytic coefficients of the hydrolysis reactions of cyclic and acyclic derivatives in this study. It is clear that *exo*-cyclic orthoester **15** is the faster reacting geminal ether of the series, hydrolysing at five-times the rate of constitutionally isomeric **8** (Figure 1). The kinetic anomeric effect was invoked previously to rationalise the relative reactivity of **15** with respect to *endo*-cyclic **8** [18]. One would anticipate a significant change in rate to accompany the reduction of charge-stabilising oxygen atoms within **8** to afford **4**; yet *k*_H+_(**8**)/*k*_H+_(**4**) *≈* 13:4. The antiperiplanar lone pair hypothesis (ALPH) proposes that the axial anomer of **4** constitutes the major conformer in solution [28], perhaps affording some stereoelectronic advantage to an early transition state which appears operative in the case of such acid-catalysed processes [29]. The stereoelectronic advantage [30] of an *endo*-cyclic oxygen atom in **4** may mitigate the loss of an electrostatically stabilising oxygen atom from the system. The hydroxonium catalytic coefficient for the hydrolysis of **1** is ≈650 times slower than observed for the constitutional isomer **4**. Acid-catalysed cleavage of the former affords a leaving group covalently tethered to a cation which renders the overall rate apparently slow, perhaps through a favoured re-cyclisation. It is noteworthy that the hydroxonium catalytic coefficient for the hydrolysis – albeit measured in water – of a similar yet acyclic ketal (i.e., 2,2-diethoxypropane) is several orders of magnitude greater than **1** [18].

### Relative reactivity of cyclic orthoesters; five versus six-membered rings

**Figure 2:**
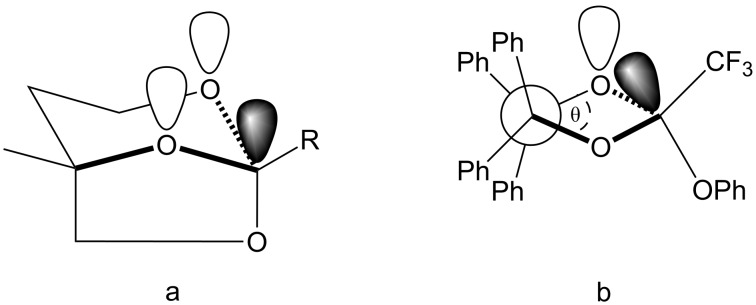
Stereoelectronic contributions to hydrolysis; (a) conformationally constrained 1,3-dioxane orthoester; (b) Newman projection of five-membered ZICMED viewed C(4)→C(5).

As *k*_1_ ≈ *k*_obs_ in the pH range examined here (Scheme 1), factors associated with the relief of cyclic strain cannot be used to account for the difference observed for five-membered **5** and six-membered **8** (i.e., *k*_H+ _*=* 6.5 and 9.8 M^−1^ s^−1^). Further, the hydroxonium catalytic coefficient for the hydrolysis of **5** and acyclic analogue **6** are within experimental error of each other (i.e., *k*_H+ _*=* 6.5 ± 0.2 and 7.0 ± 0.2 M^−1^ s^−1^, respectively). It was noted previously that the relative rates of hydrolysis for six-membered **15** and **8** could be explained with the kinetic anomeric effect. Consistent with this, the X-ray crystal structure of an analogous yet conformationally constrained bicyclic orthoester possesses an unusually elongated axial C─O bond (Figure 2a), which undergoes preferential cleavage with Lewis acids [31]. The Cambridge Structural Database (2015) [32] contains a single example of a five-membered 1,3-dioxolane orthoester [33]. Here, the ring adopts a distorted half-chair (*C*_2_) arrangement with a dihedral angle θ [O–C(4)–C(5)–O] = 32° [Figure 2a – ZICMED viewed C(4)→C(5)]. This, along with a rate of hydrolysis similar to an acyclic system suggests that a kinetic anomeric effect does not extend to 1,3-dioxolane orthoesters.

### The reactivity of C(4/5) substituted 1,3-dioxolanes

**Figure 3:**
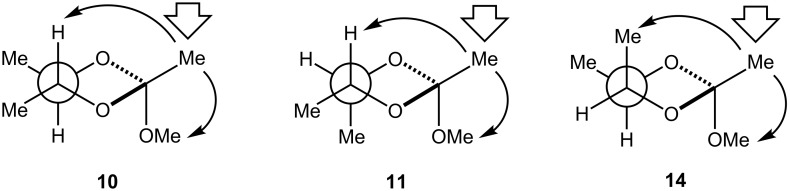
The assignment of **10**–**11** and **14** via nOe [viewed C(4)→C(5)].

Substitution of the 1,3-dioxolane ring at C(4/5) introduces asymmetry with the attendant challenges of isomer separation and identification. Here, the unambiguous assignment of the crude mix of **10**, **11** and **14** via ^1^H NMR (500 MHz) and 1D-NOESY facilitated kinetic analyses without recourse to separation. The *C*_2_ symmetry of **14** renders the C(4/5)–C*H*_3_ and C(4/5)–*H* nuclei equivalent; the methyl C(4/5)–C*H*_3_ [δ_Me_ = 1.22/1.29 ppm (d, 3H, *J =* 6.0 Hz)], and methine C(4/5)–*H* [δ_H_ = 3.73/3.83 ppm (m, 1H)] nuclei of **10** were assigned. Irradiation of the ^1^H NMR resonance associated with the C(2)–C*H*_3_ nuclei (1.46 ppm) of **10** afforded strong nOes of the multiplet at 3.73 ppm, and the singlet at 3.24 ppm, consistent with proximal *syn* C(4/5)–*H*, and the geminal C(2)–OC*H*_3_ nuclei, respectively (Figure 3). The ^1^H NMR spectra of **11** and **14** were assigned in a similar fashion.

We attribute the two-fold rate increase of **10**–**11** (*k*_H+_ ≈ 12 M^−1^ s^−1^) with respect to **5** (*k*_H+_ = 6.5 M^−1^ s^−1^) to be steric in origin. The additional two-fold rate acceleration of **14** (*k*_H+_ = 27.9 M^−1^ s^−1^) with respect to **10–11** is ascribed to the relief of transannular compression involving the C(4/5)–*Me* and *Me*–C(2) groups, which nOe studies suggest are near to each other in space (see structure **14**, Figure 3).

### The reactivity of C(2) substituted 1,3-dioxolanes

**Figure 4:**
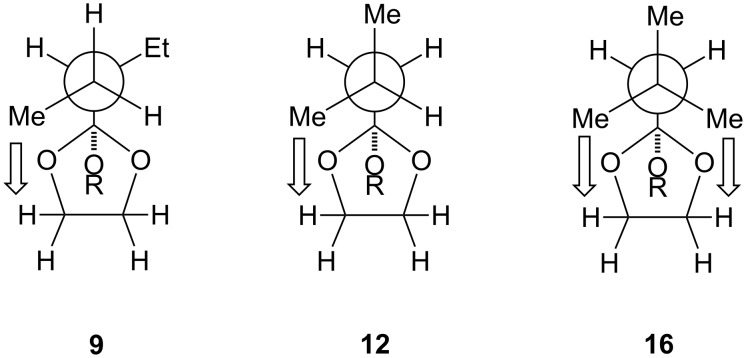
Newman projections of **9**, **12** and **16** (viewed along Cβ→Cα).

McClelland et al. [23] has determined *k*_H+_ for **5**, **7**, **9**, **12** and **16** in a water/phosphate buffer system at pH 6–7; we prepared **5** and **16** to calibrate their data with the conditions employed here (i.e*.,* D_2_O/CD_3_CN/HCl), whilst assuming that the relative magnitudes of the hydroxonium catalytic coefficients remain consistent throughout (Table 2, Experimental section). The rates of hydrolysis for **5** and **7** are essentially the same, indicating that a *tert*-butyl group at Cα exerts little or no transannular steric demand which might manifest itself in the rate determining step. In the case of Cβ substituted derivatives, a gradual increase in the hydroxonium catalytic coefficient is observed with respect to **5**, with a dramatic acceleration noted for the case of **16** (i.e., *k*_H+ _**5** = 6.5; **9** ≈ 11; **12** ≈ 14; **16** = 75.9 M^−1^ s^−1^; Figure 1). Inspection of Newman projections (Figure 4) of **9** and **12** reveal incremental 1,3-transannular steric demand associated with *Me*–Cβ and C(4/5)–*H* atoms; consistent with the gradual increase of *k*_H+_. In the case of neopentyl **16** however, two *Me*–Cβ groups are oriented toward transannular C(4/5)–*H* atoms at all times, suggesting a means by which this substituent affects a dramatic (>400%) rate increase for this substrate – this is examined further.

**Figure 5:**
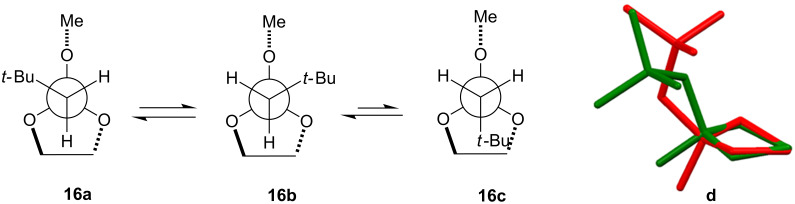
Newman projections [viewed Cα–C(2)] of the preferred *C*_2_ arrangement of the 1,3-dioxolane ring depicting the *syn* (**16a**,**b**), *anti* (**16c**) conformers, with (**d**) the superimposed calculated *syn* (**16a** = green) and *anti* (**16c** = red) structures viewed C(4)→C(5) [the C(2)O–*Me* group has been removed for clarity].

To understand the significant increase in the hydroxonium catalytic coefficient with varying C(2) substituent, computational conformational analyses of **5** and **16** were performed using Density Functional Theory to optimise the resultant structures (see Experimental section). The half-chair (*C*_2_) arrangement was found to be the only stable conformer for the 1,3-dioxolane ring with all other conformers being rotamers about the C(2)–CH_2_R and C(2)–OMe bonds [where R = H (**5**) or *t*-Bu (**16**)]. The orientation of the C(2)O–*Me* group is ignored from this point as it does not substantially affect the relative energies of the C(2)─CH_2_R rotamers. The potential energy surface for **16** is dominated by the *syn* arrangement of R with respect to the OMe group (**16a** and **16b** in Figure 5; ΔΔ*H* = 0 and 0.8 kJ/mol, respectively); the rotamer which orients the R group *anti* with respect to the OMe (**16c** in Figure 5; ΔΔ*H* = 6 kJ/mol) leads to a pseudo-axial orientation of the OMe group through flattening of the 1,3-dioxolane ring (Figure 5d); presumably this relieves steric pressure between the *t*-Bu and C(3/5)–*H* atoms at a cost of approximately 5.7 kJ/mol higher enthalpy. No such flattened conformer exists for **5**.

The calculations are consistent with the model presented earlier in Figure 4. The increasing steric demand of substituent R [i.e., R = H (**5**); → Et (**9**); → iPr (**12**) → *t-*Bu (**16**)] leads to transannular clashing with axial C(3)/(4)–*H* atoms and subsequent flattening of the 1,3-dioxolane ring. For **16** this affords an energetically accessible conformer **16c** which resembles the planar geometry anticipated for the transition state (Scheme 1), and should therefore be entropically favoured following the principle of least molecular motion. To confirm whether there was any enthalpic barrier to elimination of the protonated methoxy group, geometric scans for potential transition-states were made using Density Functional Theory calculations, by incrementally increasing and fixing the C(2)─OMe bond length for rotamers **16a**–**c** after protonation, and allowing all other geometry elements to optimise. In all cases, the five-membered ring moved towards the final planar oxonium ion, but no enthalpic barrier was found for the C(2)–OMe bond cleavage. This supports entropic control of this elimination reaction, and it is therefore not surprising that the more planar ring for the *anti* rotamer **16c** would lead to a more rapid elimination of methanol after protonation, consistent with an earlier transition state [29]. Compound **5**, which does not have such an accessible flattened ring conformation cannot access this lower entropy trajectory and hence reacts more slowly.

#### Exclusion of O(1/3)─C(5/4) cleavage

**Scheme 2:**
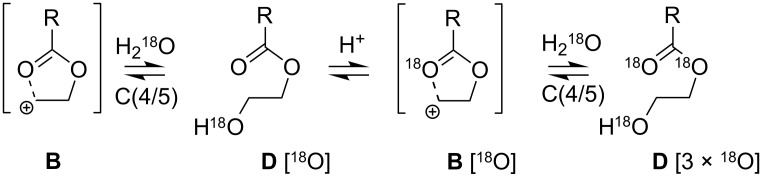
Isotopomers derived from C(4/5) hydrolytic attack of a generic 1,3-dioxolan-2-ylium cation **B** by H_2_^18^O.

It has been noted that annular strain in *trans*-2,5-dimethyltetrahydrofurans invokes sufficient charge separation to switch the mechanism of ring cleavage from *S*_N_2 → *S*_N_1 [34]. We therefore sought to exclude the possibility of mechanistic partitioning via some strain-induced O(1/3)–C(5/4) cleavage pathway for the systems under study here. Though the products of hydrolytic attack at C(2) or C(4/5) are constitutionally indistinguishable, the participation of a C(4/5) pathway brought about through intramolecular strain may be detected using H_2_^18^O labelling (Scheme 2). The C(2) attack of cation **B** by H_2_^18^O will afford **D** with ^18^O incorporated at the carbonyl carbon atom alone; acid-catalysed re-closure eliminates the heavy isotope to afford once again, **B** [35]. Alternatively, strain-induced charge separation with subsequent H_2_^18^O attack at C(4/5) affords after ring cleavage hydroxy ester **D** with ^18^O incorporated at the OH function alone; subsequent acid-catalysed re-closure affords **B** [^18^O] as isotopically distinct mesomers. Further attack of **B** [^18^O] by H_2_^18^O affords **D** [2 × ^18^O]. In short, an ambident cation **B** exposed to repetitive C(4/5) H_2_^18^O attack will ultimately afford a product incorporating ^18^O at all oxygen containing functions, i.e*.,* **D** [3 × ^18^O].

We examined **5** alongside **16** as the former, which hydrolyses at 10% the rate of the latter, is not assumed to experience any significant degree of strain. The 1,3-dioxolan-2-ylium salts derived from **5** (5*, i.e., **B** R = Me) and **16** (16*, i.e*.,* **B** where R = *n*-Pent) were prepared via an unambiguous route [36–38] and quenched using H_2_^16^O or H_2_^18^O and analysed via MS(CI). The [M + H]^+^ and [(M + 2) + H]^+^ ions derived from the reaction of 5* and 16* with H_2_^16/18^O alone were evident, indicating the incorporation of a single ^18^O atom. To establish the site of isotope incorporation, crude mixtures were examined via ^13^C NMR which identified in both cases a characteristic upfield shift (Δδ_C_ = 0.04 ppm) of the resonance attributed to the carbonyl carbon atom [39–40], consistent with C(2) attack alone. We confirm therefore that the dramatic rate acceleration noted for **16** is not consistent with a change in mechanism [41].

## Conclusion

A range of promising FDII candidates based upon acyclic and cyclic geminal ethers, unambiguously organised according to their catalytic coefficients *k*_H+_ in CD_3_CN/D_2_O 4:1 has been presented (Figure 1). As anticipated, orthoacetates possessing ethoxy substituents are the most rapidly hydrolysed acyclic systems. Both entropic and steric effects are believed to account for the relatively fast rates of hydrolysis of cyclic orthoesters with respect to ketals. Rate increases within five-membered *endo*-cyclic orthoesters accompany increasingly bulky C(2) alkyl substitution. Dramatic increases in the hydroxonium catalytic coefficients for hydrolysis are observed for Cβ branching at C(2), which can lead to conformational distortion of the five-membered ring which lowers the activation barrier to elimination of the OMe group and formation of the intermediate oxonium ion. The dual performance of cyclic geminal ethers as FDII for jet fuels will be reported shortly.

## Experimental

All preparative operations were performed at the synthetic laboratories of the School of Chemistry, University of Bristol. NMR spectra were recorded on a JEOL ECP Eclipse 300 spectrometer at 300 MHz (^1^H), a JEOL ECP Eclipse 400 spectrometer at 400 MHz (^1^H) and 100 MHz (^13^C) or a Varian VNMRS 500 spectrometer at 500 MHz (^1^H) in deuterated solvents (University of Bristol). Mass spectra (HRMS) were obtained on a Micromass AutoSpec MS in CI mode using CH_4_ as the collision gas. HRMS and elemental analysis were performed by the respective services at the School of Chemistry, University of Bristol.

**Orthoester hydrolysis kinetics.** The reacting pair of **2** and **6** has been used here to illustrate a typical procedure. Adjustments to the acid catalyst concentration were made on an iterative basis in order to ensure the kinetic runs were complete within reasonable time-scales. Freshly distilled **2** (33 µL, 0.3 mmol) and **6** (38 µL, 0.3 mmol) were added to anhydrous CD_3_CN (1200 µL), and the resulting solution was divided equally into three dry 5 mm NMR tubes. ^1^H NMR (300 MHz) spectroscopy was used to confirm the resulting 1:1 ratio of **2** and **6** in each sample. To minimise contamination by H_2_O, acid solutions were prepared by the successive dilution of HCl (35% v/v) with D_2_O. In this manner, a solution of [H^+^] = 4.78 × 10^–4^ M was prepared; [H^+^] was confirmed via the measurement of [Cl^−^] using ion chromatography. The hydrolysis reaction was initiated by the addition of HCl in D_2_O (100 µL, 4.78 × 10^−8^ mol) to the NMR tube. Data was acquired every 625 s until the faster of the pair was consumed. NMR kinetic measurements were temperature-controlled at 25 ± 0.5 °C using the variable temperature control unit of the spectrometer (instrument temperature calibration performed with an internal NMR methanol thermometer). A semi-logarithmic plot of the integral of the resonances associated with **2** (δ_H_ = 4.93 ppm), and **6** (δ_H_ = 1.35 ppm) against time (s) affords two straight lines with gradient = *k*_obs_, which when divided by [H^+^], provides the catalytic coefficient *k*_H+_. The process was repeated a further two times using the pre-prepared samples. The value of *k*_H+_ remains constant throughout the range [H^+^] ≈ 5 → 10 × 10^−5^ M, thereby confirming that it is only necessary to measure *k*_obs_ for a given value of [H^+^] in order to determine *k*_H+_ (Table 1).

**Table 1:** Values of *k*_obs_ and *k*_H+_ for **2** and **6** at different acid concentrations.

[HCl] × 10^−5^ M	*k*_obs_ × 10^−6^ s^−1^		*k*_H+_ M^−1^ s^−1^	

	**2**	**6**	**2**	**6**

9.56	14.6	678	0.150	7.09
6.38	9.4	447	0.147	7.01
5.31	7.7	363	0.145	6.84

### Calibration of rate data

**Table 2:** The ratio of the rates of hydrolysis for **1**, **4**, **8** and **15**.

	Rate ratios^a^	*k*_H+_ (M^−1^ s^−1^)^b^

**1**	1	≈4 × 10^-3^
**4**	649	≈3
**8**	2270	9.8 ± 0.2
**15**	11351	≈49

^a^Determined in D_2_O/CD_3_CN (1:4 v/v) as reported by Deslongchamps et al. [18], where the relative reaction rates were determined across a range of [H^+^] (errors not reported in original work). ^b^Values of *k*_H+_ estimated through calibration with the experimentally determined value of **8** established by this work.

**Table 3:** Calibrating *ratios* of *k*_1_^H^ for **5**, **7**, **9**, **12** and **16** with respect to experimentally determined values of **5** and **16** from this study.

	*k*_1_^H^ ratio^a^	*k*_H+_ (M^−1^ s^−1^)^b^	*k*_H+_ (M^−1^ s^−1^)^c^	*k*_H+_ (M^−1^ s^−1^)^d^

**5**	1	6.5 ± 0.2	–	6.5 ± 0.2
**6**	–	–	–	7.0 ± 0.2
**7**	2.0	≈13	≈8	–
**9**	2.6	≈17	≈11	–
**12**	3.4	≈22	≈14	–
**16**	18.8	≈123	≈76	75.9 ± 7.1

^a^The *ratio* of *k*_1_^H^ (equivalent to *k*_H+_ in this work) determined in water/phosphate buffer are considered here as we believe that [23] contains typographical errors in the reported exponential factors. ^b^Calibration of *k*_1_^H^ ratios with respect to the experimentally determined value of **5**^d^ then **16**^c^ (× 76/123). For consistency, **6** and **5** were paired for kinetic runs.

**Preparations.** The acyclic orthoesters **2**, **3**, **6** and **13** are commercially available, whereas the cyclic derivatives **5** [23,42], **8** [43–44], **10** [43], and **16** [23,36,45] were prepared via known procedures. Though mixtures of **10**, **11** and **14** have been prepared previously [46–47], **11** and **14** have not been characterised. Thus, freshly distilled **6** (9.3 mL, 73 mmol) was added with stirring to a pre-cooled (0 °C) solution of 2,3-butanediol (6.6 g, 73 mmol), and H_2_SO_4_ (100 µL, 2.4 mmol) in diethyl ether (30 mL) under nitrogen. The reaction was quenched after 24 hours by the addition of imidazole (0.4 g, 6 mmol), and the resulting cloudy solution was washed with saturated aqueous NaHCO_3_ (200 mL), filtered and extracted with diethyl ether (3 × 50 mL). The combined organic extracts were dried (MgSO_4_), and concentrated in vacuo to afford a clear colourless liquid characterised (via 1D NOESY ^1^H NMR spectroscopy) as a mixture of **10** (30%), **11** (20%) and **14** (50%) (6.9 g, 65%). Repeated distillations (37 °C, 10 mmHg) failed to afford separation, and column chromatography (SiO_2_, petroleum ether/ethyl acetate/triethylamine 15:4:1) resulted in the hydrolysis of products. δ_H_ (**10**) [43] (500 MHz, CDCl_3_) 1.25 (d, *J* = 6.0 Hz, 3H), 1.30 (d, *J* = 6.0 Hz, 3H), 1.53 (s, 3H), 3.29 (s, 3H), 3.72 (m, 1H), 3.84 (m, 1H); δ_H_ (**11**) (500 MHz, CDCl_3_) 1.22 (m, 6H), 1.50 (s, 3H), 3.30 (s, 3H), 4.29 (m, 2H); δ_H_ (**14**) (500 MHz, CDCl_3_) 1.15 (m, 6H), 1.56 (s, 3H), 3.28 (s, 3H), 4.41 (m, 2H). δ_C_ (**10**, **11**, **14**) (100 MHz, CDCl_3_) 15.3, 15.5, 17.0, 17.4 [C(4/5)-*C*H_3_)], 22.5, 23.3, 23.6 [C(2)-*C*H_3_], 49.3*, 50.5 (-O*C*H_3_), 73.9, 74.6, 79.1, 79.6 [*C*(4/5)], 120.0, 121.0 [*C*(2)]. *Signal splitting of Δδ = 0.09 ppm observed. HRMS–ESI calculated for [M + Na]^+^ 169.0835, found: 169.0836.

The distribution of products resulting from the exchange reaction of **6** with *dl-* and *meso*-2,3-butanediol warrants brief comment; reaction of the former affords **10** [43], whereas the latter gives C(2) epimers **11** and **14**. Both GC–MS and ^1^H NMR analyses (see Supporting Information File 1) of the crude generated by treatment of **6** with a 1:1 mixture of *dl*/*meso*-2,3-butanediol indicates **10**/**11**/**14** are formed in the ratio 3:2:5, respectively (63% conversion). Yet treatment of **6** with commercially available 2,3-butanediol (77:23 *meso-* and *dl-,* respectively by ^1^H NMR [48]) also affords the same product ratio with 65% conversion. Performing the procedure at −10 and +20 °C does not change the product distribution; we conclude then, that the exchange reaction proceeds via equilibrium control.

**Computational techniques***.* X-ray crystal structures were located in the 2014 release of the Cambridge Structural Database (CSD v 5.35, which contains 658, 007 entries) using the Conquest software (v 1.16) and visualised using the Mercury software package (v 3.1). Conformational searching was conducted with Macromodel software, using a MonteCarlo search method and the MMFF force field. Final molecular geometries were optimised using the Gaussian09 [49] with density functional level of theory, using the hybrid functional B3LYP/6-31G* to optimise structures [50–52], with convergence criteria for maximum and RMS force (0.000450 and 0.000300 in atomic units per Bohr and per radian respectively) and for atomic displacements (0.001800 and 0.001200 Angstroms, respectively).

## Supporting Information

Supporting Information features copies of an indicative ^1^H NMR stacked plot (**2**/**6**), 1D NOESY spectra (**10**/**11**/**14**), MS(CI) spectra (5*/16*), experimentally determined values of *k*_H+_ for the reacting mixtures of geminal ethers: [**6** + **2**], [**3** + **2**], [**6** + **13**], [**6** + **5**], [**6** + **8**], [**6** + **10** + **11** + **14**], [**6** + **16**], and Cartesian coordinates of conformers **16c**.

File 1 Analytical data.
