# Supplementary material for: The hydrolysis of geminal ethers: a kinetic appraisal of orthoesters and ketals
Source: Beilstein J Org Chem. 2016 Jul 15;12:1467–75. doi: 10.3762/bjoc.12.143 (PMC4979634; doi:10.3762/bjoc.12.143)
Supplement: File 1 — Analytical data. [file Beilstein_J_Org_Chem-12-1467-s001.pdf]

Supporting Information

for

The hydrolysis of geminal ethers: a kinetic appraisal of orthoesters and ketals

Sonia L. Repetto[1], James F. Costello*[1], Craig P. Butts[2], Joseph K. W. Lam[3] and Norman M. Ratcliffe[1]

Address: [1]Faculty of Applied Sciences, University of the West of England, Bristol, BS16 1QY, UK, [2]School of Chemistry, University of Bristol, Bristol, BS8 1TS, UK and [3]Airbus Operations Ltd., Filton, Bristol, BS34 7PA, UK

Email: James F. Costello - James.Costello@uwe.ac.uk

*Corresponding author

Analytical data

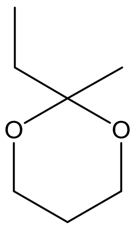

1

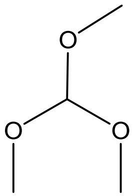

2

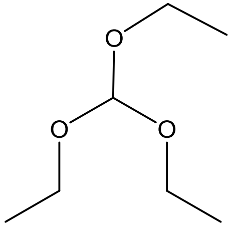

3

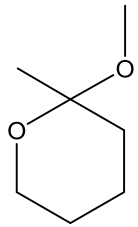

4

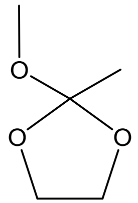

5

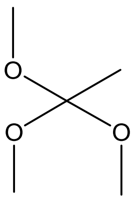

6

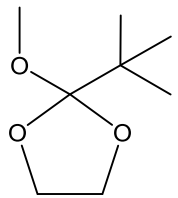

7

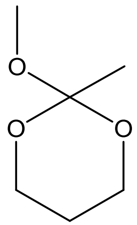

8

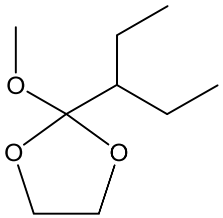

9

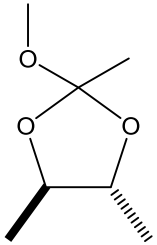

10

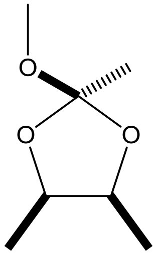

11

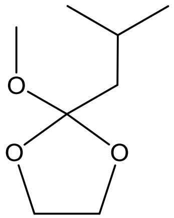

12

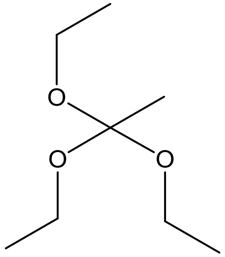

13

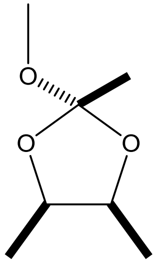

14

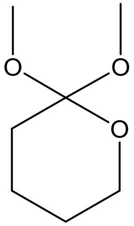

15

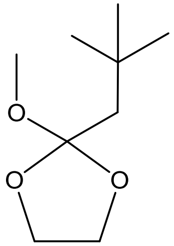

16

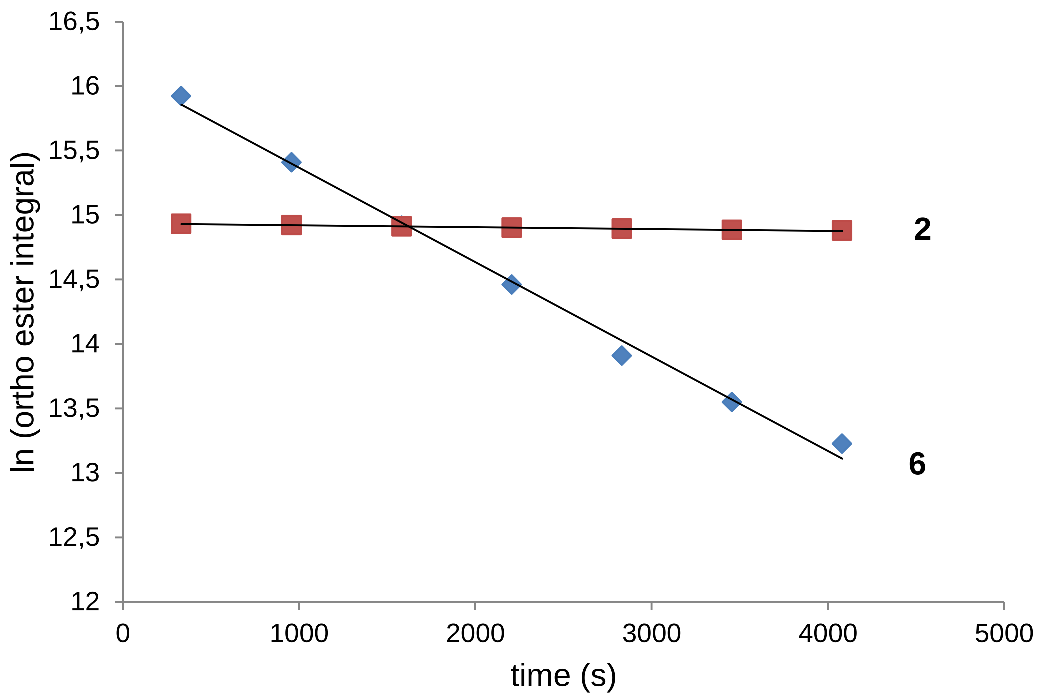

Indicative plot of ln(integral) for **6** (k_{obs} = 7 × 10^{-4}; R^2 = 0.9944), and **2** (k_{obs} = 1.46 × 10^{-5}; R^2 = 0.9932), versus time (s).

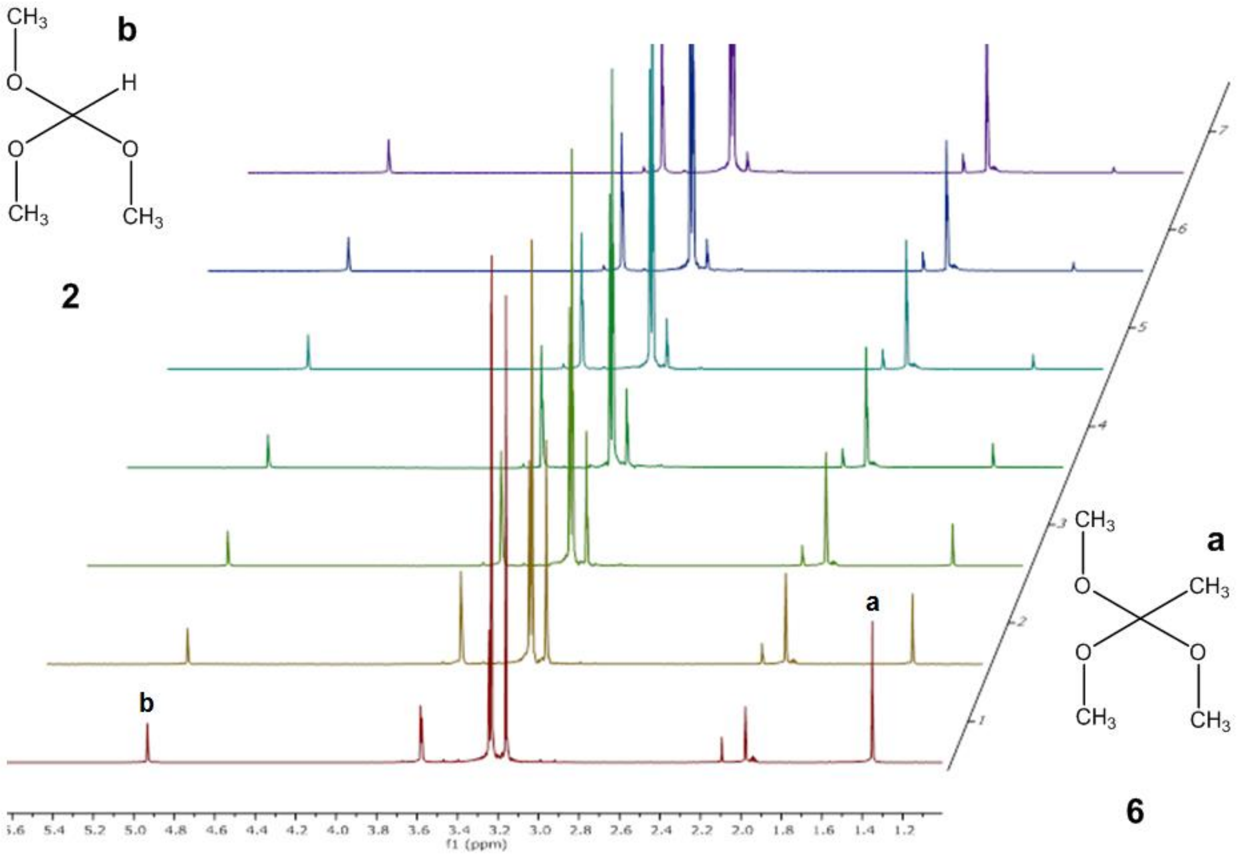

Indicative stacked plot of **6** and **2** versus time (300 MHz ^{1}H NMR in D_2O/CD_3CN (1:4), 625 s intervals, *T*= 25 °C, [HCl] = 9.56 x 10^{-5} M).

Table S1: The concentration of acid catalyst used to determine values of k_{H+} for the following mixtures of reacting ortho esters.

Reacting substrates	$[H^+] \times 10^{-4}$ M
**6:2**	4.78
**3:2**	4.78
**6:13**	1.98
**6:5**	1.98
**6:8**	1.98
**6:10:11:14**	1.98
**6:16**	0.125

Table S2: Experimentally determined values of k_{H+} for the reacting mixtures of geminal ethers: [**6** + **2**], [**3** + **2**], [**6** + **13**], [**6** + **5**], [**6** + **8**], [**6** + **10** + **11** + **14**], [**6** + **16**].

The corresponding values of k_{H+} (including standard deviations) in Figure 1 (main article) were calculated from an average of these experiments. Thus, k_{H+} for **5** calculated using entries (x–xii). Also, k_{H+} for **6** calculated from entries (i–iii), (vii–ix), and (x–xxi) respectively, along with data from Table 1 of main article.

	**6**	**2**
(i)	7.32	0.153
(ii)	6.74	0.145
(iii)	7.20	0.151

	**3**	**2**
(iv)	1.650	0.147
(v)	1.675	0.155
(vi)	1.730	0.143

	**6**	**13**
(vii)	6.91	19.44
(viii)	7.20	19.74
(ix)	6.89	21.18

	**6**	**5**
(x)	7.50	6.70
(xi)	6.70	6.30
(xii)	7.20	6.60

	**6**	**8**
(xiii)	6.84	9.77
(xiv)	6.99	10.00
(xv)	6.52	9.57

	6	10	11	14
(xvi)	7.07	13.10	13.99	29.80
(xvii)	7.01	11.76	12.78	27.38
(xviii)	6.90	11.38	12.07	26.62

	6	16
(xix)	7.00	70.63
(xx)	7.20	73.10
(xxi)	6.74	84.00

Table S3: Corresponding values of k_{H+} ($M^{-1}s^{-1}$) obtained from calibration of data obtained from different workers.

Ortho ester	Relative rate[a]	k_{H+} ($M^{-1}s^{-1}$)
**1**	1	4 x 10^{-3}[b]
**4**	649	2.79[b]
**8**	2270	9.78[c]
**15**	11351	48.90[b]

Ortho ester	k_{1H+} ratios[a]	k_{H+} ($M^{-1}s^{-1}$)[b]	k_{H+} ($M^{-1}s^{-1}$)[c]	k_{H+} ($M^{-1}s^{-1}$)[d]
**5**	1	6.53	–	6.5 ± 0.2
**6**	–	–	–	7.0 ± 0.2
**7**	2.0	≈ 13	≈ 8	–
**9**	2.6	≈ 17	≈ 11	–
**12**	3.4	≈ 22	≈ 14	–
**16**	18.8	≈ 123	≈ 76	75.9 ± 7.1

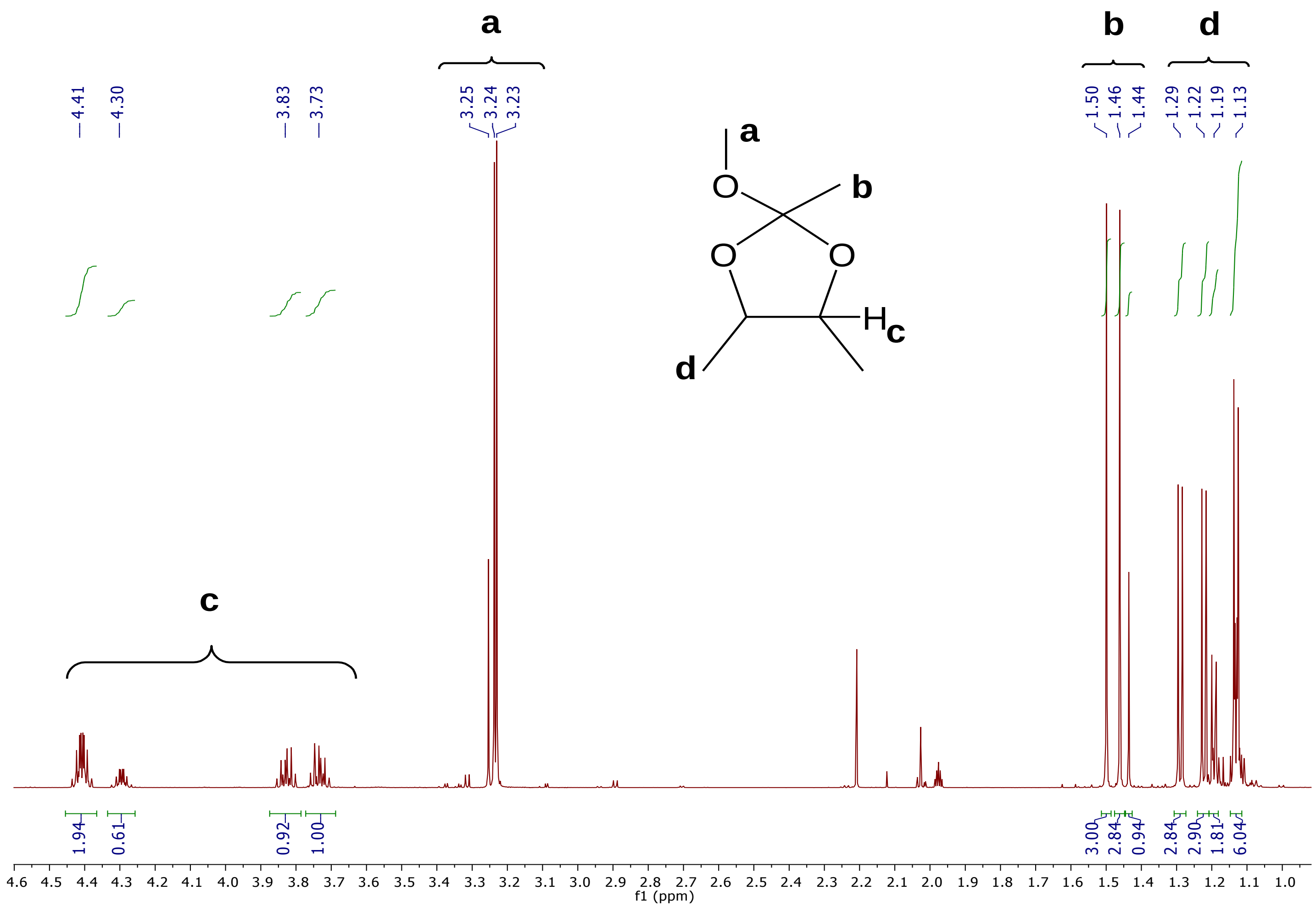

500 MHz ^{1}H NMR spectrum of a mixture of **10, 11** and **14** in CD_3CN.

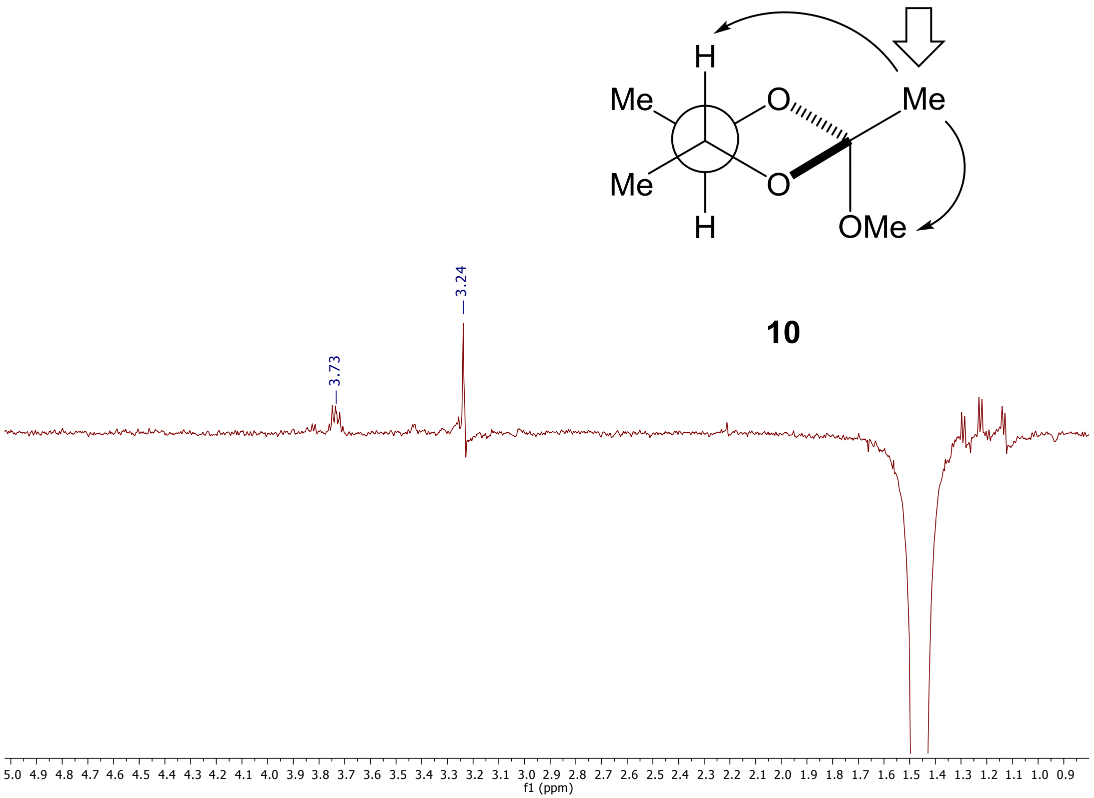

1D-NOESY ^{1}H NMR spectrum of a mixture of **10, 11** and **14** (3:1:3.2, respectively) irradiated at 1.46 ppm.

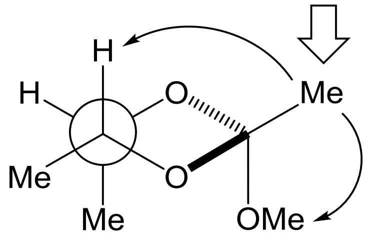

11

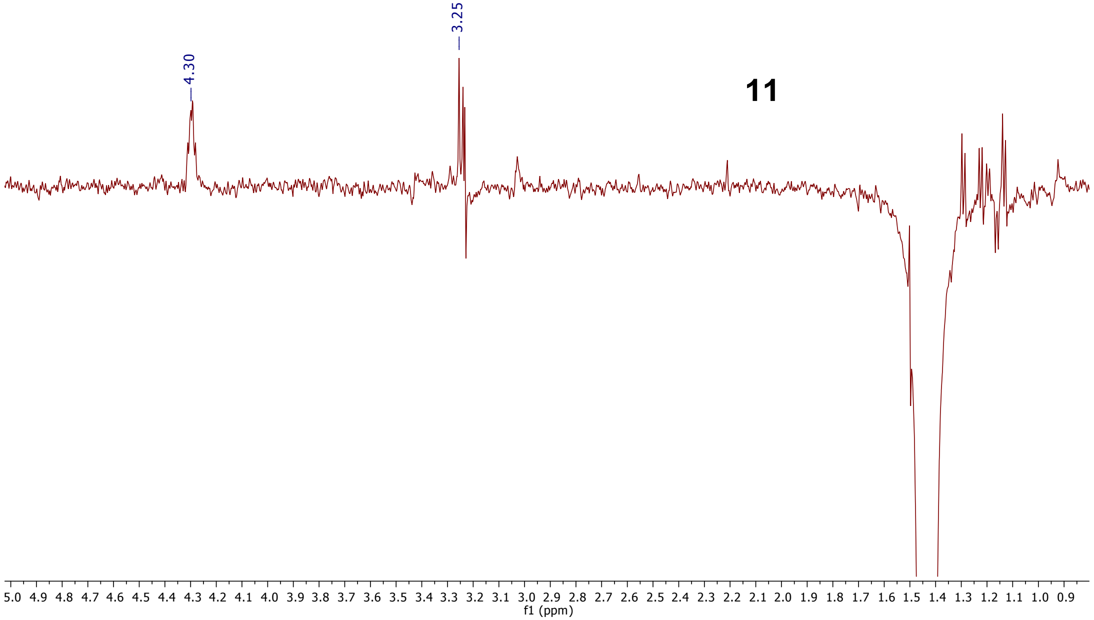

1D-NOESY ^{1}H NMR spectrum of a mixture of **10, 11** and **14** (3:1:3.2, respectively) irradiated at 1.44 ppm.

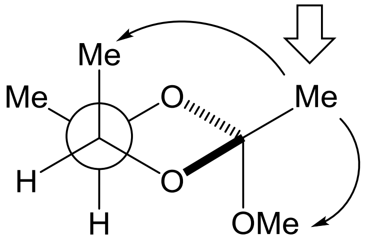

14

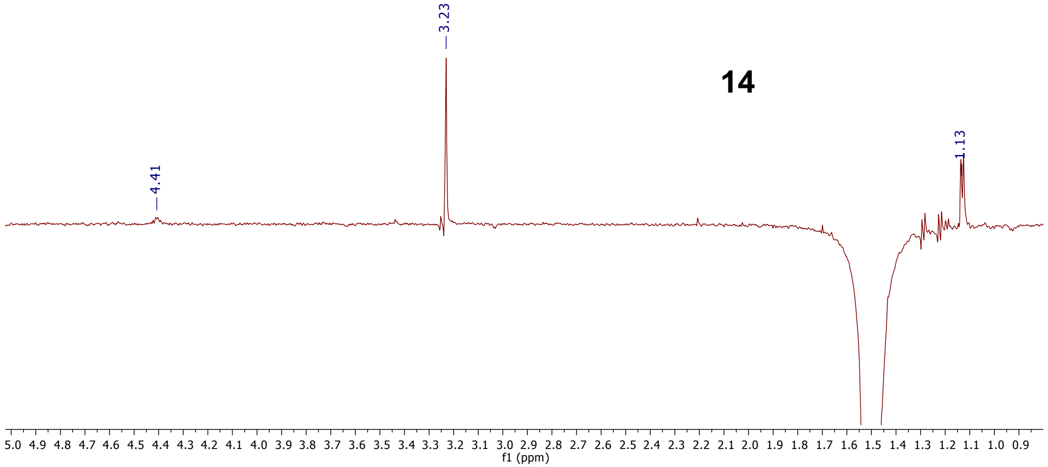

1D-NOESY ^{1}H NMR spectrum of a mixture of **10, 11** and **14** (3:1:3.2, respectively) irradiated at 1.50 ppm.

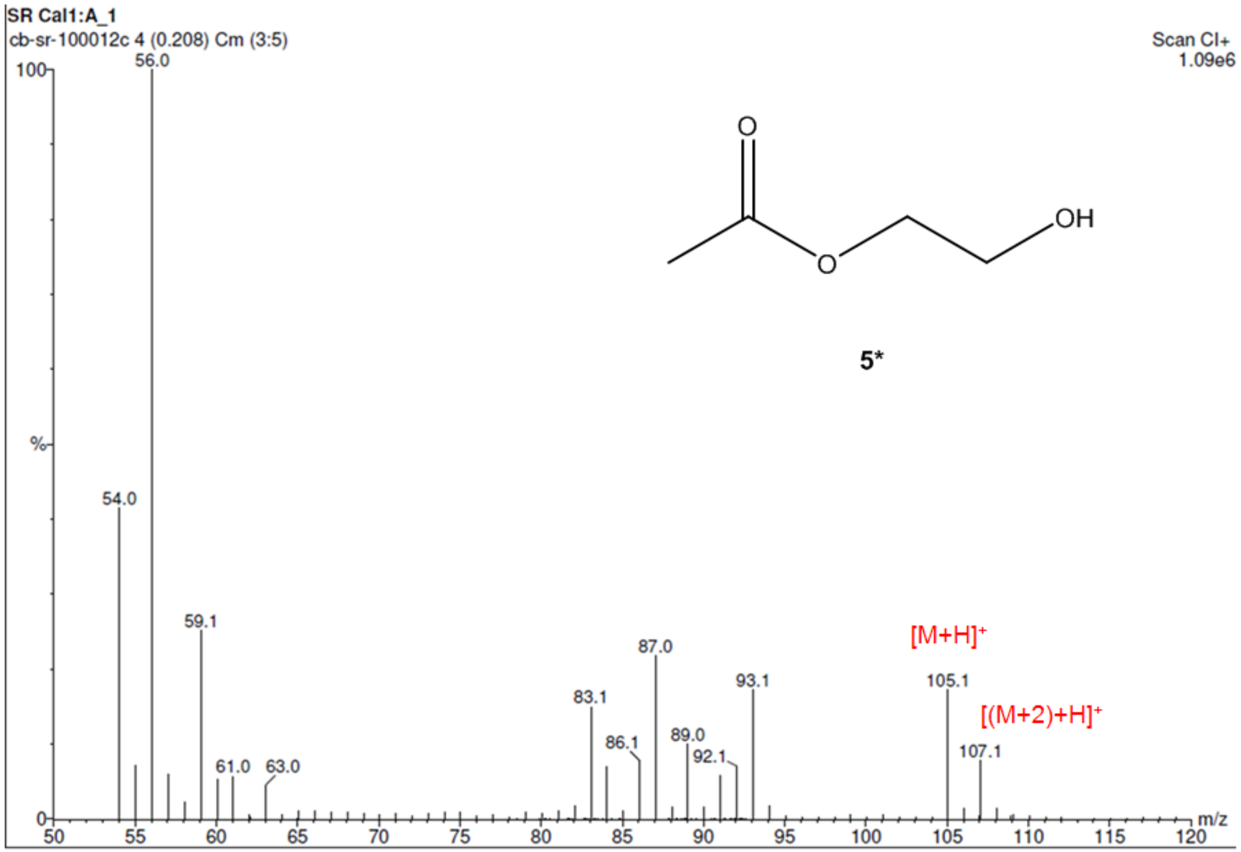

MS(CI) spectrum of hydroxy ester the derived from 1,3-dioxolan-2-ylium cation **5*** and $H_2{}^{16/18}O$.

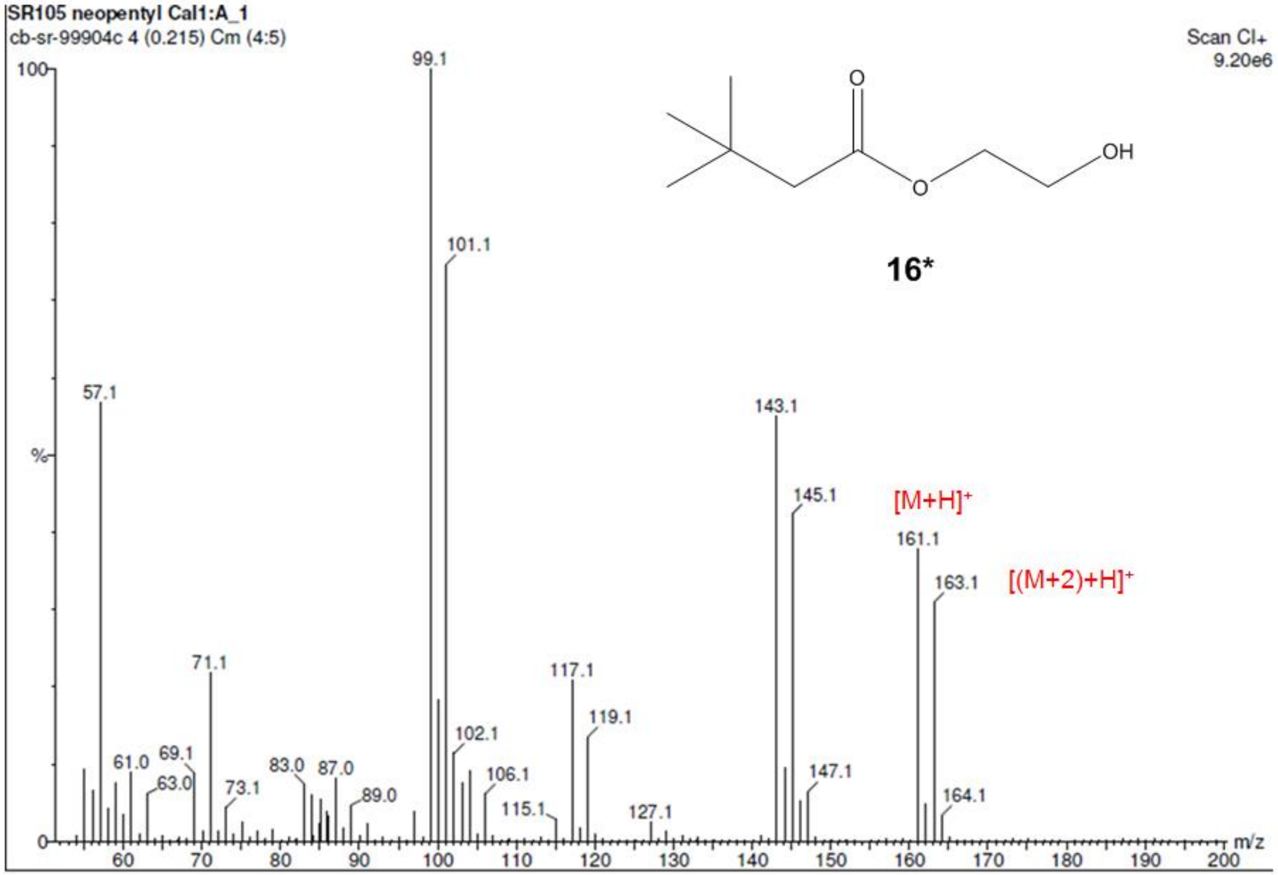

MS(CI) spectrum of the hydroxy ester derived from 1,3-dioxolan-2-ylium cation **16*** and $H_2{}^{16/18}O$.

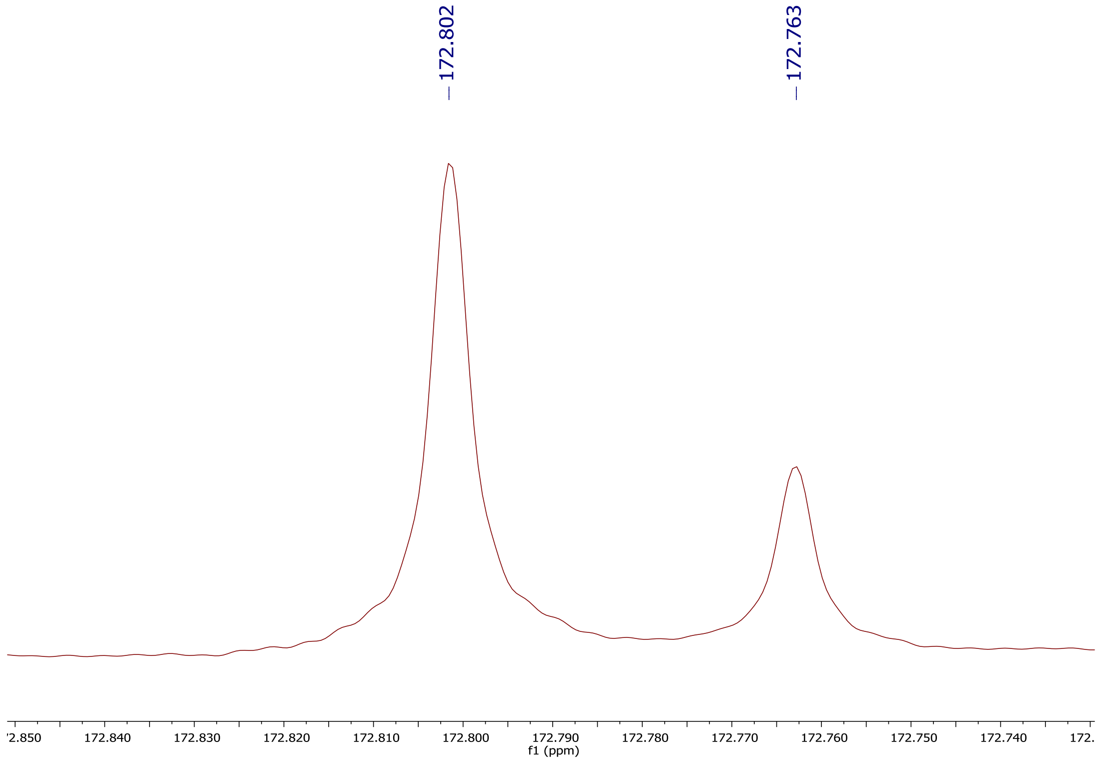

Partial ^{13}C NMR spectrum (125 MHz, $CDCl_3$) of hydroxy ester derived from **16** illustrating resonances associated with $^{13}C{=}^{18}O$ (δ_C = 172.76ppm) and $^{13}C{=}^{16}O$ (δ_C = 172.80 ppm *i.e.,* $\Delta\delta_C$ = 0.04 ppm) nuclei.

Full literature reference for Gaussian 09

Gaussian 09, Revision A.1, M. J. Frisch, G. W. Trucks, H. B. Schlegel, G. E. Scuseria, M. A. Robb, J. R. Cheeseman, G. Scalmani, V. Barone, B. Mennucci, G. A. Petersson, H. Nakatsuji, M. Caricato, X. Li, H. P. Hratchian, A. F. Izmaylov, J. Bloino, G. Zheng, J. L. Sonnenberg, M. Hada, M. Ehara, K. Toyota, R. Fukuda, J. Hasegawa, M. Ishida, T. Nakajima, Y. Honda, O. Kitao, H. Nakai, T. Vreven, J. A. Montgomery, Jr., J. E. Peralta, F. Ogliaro, M. Bearpark, J. J. Heyd, E. Brothers, K. N. Kudin, V. N. Staroverov, R. Kobayashi, J. Normand, K. Raghavachari, A. Rendell, J. C. Burant, S. S. Iyengar, J. Tomasi, M. Cossi, N. Rega, J. M. Millam, M. Klene, J. E. Knox, J. B. Cross, V. Bakken, C. Adamo, J. Jaramillo, R. Gomperts, R. E. Stratmann, O. Yazyev, A. J. Austin, R. Cammi, C. Pomelli, J. W. Ochterski, R. L. Martin, K. Morokuma, V. G. Zakrzewski, G. A. Voth, P. Salvador, J. J. Dannenberg, S. Dapprich, A. D. Daniels, Ö. Farkas, J. B. Foresman, J. V. Ortiz, J. Cioslowski, and D. J. Fox, Gaussian, Inc., Wallingford CT, 2009.

Cartesian coordinates of conformer 16a

C	-2.03182000	-1.48965000	0.70785400
C	-0.65026000	0.10995800	-0.22370600
C	-2.71696200	-0.93745800	-0.54025600
H	-1.53976800	-2.45194700	0.50530200
H	-2.69624900	-1.60348600	1.56830100
H	-3.55058500	-0.27027400	-0.28049700
H	-3.08107900	-1.71225000	-1.22112000
O	-1.07539300	-0.48067000	1.00366400
O	-1.66994800	-0.21260100	-1.18009900
C	2.01132200	-0.13827700	-0.02606300
C	-1.70594500	2.18082800	0.31258800
C	0.67229700	-0.46586200	-0.74446500
H	0.74833500	-0.16286400	-1.79470500
H	0.52920700	-1.55318900	-0.74953900
C	3.06375700	-1.11795600	-0.58936700
H	2.80847500	-2.15785700	-0.35469200
H	3.14739800	-1.03077900	-1.67892100
H	4.05146400	-0.91215200	-0.16254300
C	1.91375000	-0.33679700	1.49828200
H	2.89471900	-0.17794900	1.96086300
H	1.20598200	0.36262600	1.94880800
H	1.58261300	-1.34987200	1.74977800
C	2.47793900	1.29981200	-0.33099000
H	2.57498300	1.46087300	-1.41114800
H	1.77454100	2.03795400	0.05597000
H	3.45981200	1.48242600	0.12108900
O	-0.52029200	1.47759800	-0.05540900
H	-2.10822200	1.81140500	1.26113000
H	-2.46727000	2.09899700	-0.47042200
H	-1.41375100	3.22613200	0.42382600

Cartesian coordinates of conformer 16b

C	2.38968800	-1.37618000	-0.58742300
C	0.65369300	0.12175600	-0.27113300
C	2.27342700	-1.08285900	0.90637300
H	1.91707900	-2.33428600	-0.84817000
H	3.41693000	-1.37254000	-0.96145000
H	3.08183200	-0.42462900	1.25333400
H	2.25165200	-1.97980500	1.53181300
O	1.68860200	-0.28655300	-1.17061100
O	1.01185700	-0.42378900	1.00060800
C	-2.01088200	-0.13476500	0.00804900
C	1.76908600	2.17713900	0.20309800
C	-0.69264300	-0.42218600	-0.76409500
H	-0.56063700	-1.50891700	-0.83061300
H	-0.80009600	-0.05595300	-1.79119400
C	-1.93898400	-0.60490100	1.47351100
H	-1.67068700	-1.66512400	1.53981700
H	-1.19613600	-0.04224300	2.04237900
H	-2.91421100	-0.47521200	1.95722100
C	-3.11714400	-0.93625700	-0.71278700
H	-4.08823900	-0.77068900	-0.23356100
H	-3.20829200	-0.63589500	-1.76296700
H	-2.91244300	-2.01286900	-0.68825700
C	-2.38886300	1.35911000	-0.03438000
H	-1.66131100	1.97239700	0.49890100
H	-2.43886200	1.72540400	-1.06596300
H	-3.37390600	1.51031000	0.42230100
O	0.59430000	1.50544700	-0.24707400
H	2.63111000	1.93027200	-0.42475100
H	1.98820100	1.92961300	1.24752000
H	1.55567700	3.24418000	0.12297800

Cartesian coordinates of conformer 16c

C	1.89106600	-1.62860600	-0.60508300
C	0.70638100	0.24111600	-0.17460500
C	2.00325600	-1.31263500	0.90460600
H	1.68891600	-2.68057200	-0.81852900
H	2.78172900	-1.30766800	-1.15468900
H	3.02099100	-1.02455900	1.18911700
H	1.67908700	-2.14381600	1.53859600
O	0.75043600	-0.87067000	-1.01760200
O	1.09432100	-0.22422200	1.10544500
C	-1.95450000	0.02730500	0.03190700
C	2.00317400	2.26227600	0.12712600
C	-0.67408400	0.89747500	-0.11627700
H	-0.76624600	1.50331000	-1.02443600
H	-0.63111200	1.59724500	0.72578800
C	-3.10852700	0.99775800	0.36487900
H	-2.94503100	1.49856400	1.32609400
H	-3.21411600	1.77242400	-0.40361600
H	-4.06057800	0.45932900	0.42632800
C	-1.83563600	-1.00419600	1.16910700
H	-2.79635200	-1.51176700	1.31362500
H	-1.08407200	-1.76409400	0.94337400
H	-1.55599000	-0.52978700	2.11497200
C	-2.29071700	-0.69270300	-1.28985000
H	-2.42633500	0.02729200	-2.10541700
H	-1.49754600	-1.38314400	-1.58032100
H	-3.22472600	-1.25723200	-1.18760200
O	1.69011400	1.13564200	-0.68186000
H	2.18922200	1.97067500	1.16665100
H	1.20940700	3.02082400	0.10491700
H	2.91130600	2.70023100	-0.29357500
